# Assessment of Health-Related Quality of Life in Pediatric Acute Lymphoblastic Leukemia Survivors: Perceptions of Children, Siblings, and Parents

**DOI:** 10.4274/tjh.galenos.2018.2018.0351

**Published:** 2019-05-03

**Authors:** Deniz Kızmazoğlu, Seher Sarı, Melike Evim Sezgin, Arzu Kantarcıoğlu, Özlem Tüfekçi, Fatma Demir Yenigürbüz, Birol Baytan, Şebnem Yılmaz, Adalet Meral Güneş, Hale Ören

**Affiliations:** 1 Dokuz Eylül University Faculty of Medicine, Department of Pediatric Hematology, İzmir, Turkey; 2 Uludağ University Faculty of Medicine, Department of Pediatric Hematology, Bursa, Turkey

**Keywords:** Acute lymphoblastic leukemia, Health-related quality of life, KINDLR questionnaire

## Abstract

**Objective:**

We investigated the health-related quality of life (HRQL) in survivors of pediatric acute lymphoblastic leukemia (ALL) and evaluated the perceptions of the children, their siblings, and their parents.

**Materials and Methods:**

Seventy ALL survivors, who were between 7 and 17 years of age and had completed therapy ≥2 years, were included. The control group consisted of their healthy siblings. HRQL was assessed by the age-specific KINDL^R^ questionnaire.

**Results:**

No significant differences could be found among HRQL scores of ALL survivors with respect to variables such as sex, risk group, and having chronic illness. HRQL scores for physical well-being, emotional well-being, family, and social functioning of the patient and sibling self-reports and parent proxy reports were lower than the expected values for healthy and chronically ill children.

**Conclusion:**

These results demonstrate that both ALL survivors and their families need help via psychological counseling programs to improve their HRQL even after completion of therapy.

## Abstract (tr)

**Amaç:**

Çocukluk çağı akut lenfoblastik lösemi (ALL) sağ kalanlarında sağlıkla ilişkili yaşam kalitesinin (HRQL) araştırılması ve çocukların, kardeşlerinin ve ebeveynlerinin bununla ilgili algılamalarının değerlendirilmesi amaçlandı.

**Gereç ve Yöntemler:**

Çalışma grubu 7-17 yaş arası, kemoterapisi en az 2 yıl önce tamamlanmış 70 ALL’li çocuktan oluşturuldu. Kontrol grubu hastaların sağlıklı kardeşlerini (n=32) içerdi. HRQL ölçümü için yaşa uygun KINDL^R^ anket formları kullanıldı.

**Bulgular:**

ALL sağ kalanlarında HRQL skorları cinsiyet, risk grubu, kronik hastalığı olma ve relaps öyküsü açısından istatistiksel anlamlı fark göstermedi. Hastalar ve kardeşlerinin HRQL skorlarıyla ebeveynlerin hasta çocuklarının durumuna bakış açısı skorları değerlendirildiğinde fiziksel iyilik, duygusal iyilik, aile ve sosyal ilişkiler ile ilgili HRQL skorları sağlıklı ve kronik hastalığa sahip çocuklara göre düşük bulundu.

**Sonuç:**

Hasta ve ebeveynlerin HRQL skorlarının düşük saptanması çocukluk çağı ALL sağ kalanları ve ebeveynlerinin tedavi bitimi sonrasında da psikolojik danışmanlık programlarına ihtiyacı olduğunu göstermektedir.

## Introduction

Acute lymphoblastic leukemia (ALL) comprises 26%-28% of all childhood malignancies [1,2]. The outcome of ALL has improved and survival periods have become longer [3,4]. Five-year overall survival was 79.9% at our centers between 1995 and 2009 [5]. Investigations of survivors have documented that their quality of life is negatively affected, with difficulties in social and intellectual functioning [6,7,8,9,10].

The aim of this study was to investigate health-related quality of life (HRQL) in survivors of pediatric ALL and to evaluate the perceptions of the children, their siblings, and their parents.

## Materials and Methods

### Patients

The study population consisted of children who were diagnosed with ALL and had been treated with the ALL Berlin-Frankfurt-Muenster (BFM) 95 protocol [11] at Dokuz Eylül University and Uludağ University Hospitals. We included 70 children diagnosed with ALL who were between the ages of 7 and 17 years, in complete remission, who had been followed for ≥2 years after completion of therapy. The control group consisted of 32 healthy siblings. We analyzed only one sibling of a patient who was in the age group of 7-17 years to obtain reliable results, since children may begin adequately describing their HRQL by the age of 7 years [12]. One parent for each patient (62 mothers, 8 fathers) filled out a questionnaire.

The sociodemographic data of the patients were collected via questionnaire. Data about ALL therapy and follow-up period were recorded from hospital files. If the family’s monthly income was under 2000 Turkish lira (TL), participants were classified in the lower income group; if it was between 2000 and 5000 TL, they were classified in the middle income group; and if it was above 5000 TL, they were classified in the higher income group.

### Evaluation of HRQL

There are different methods for evaluating HRQL [12,13,14,15,16]. We used the KINDL^R^ questionnaire for measuring HRQL in children and adolescents. The KINDL^R ^questionnaire was developed by Bullinger et al. and revised by Ravens-Sieberer and Bullinger [13,14,15,16,17]. The KINDL^R ^aims more at the psychosocial than the physical aspects of HRQL. The method of assessment is child self-report or parent proxy report. Age- and sex-specific versions take into account the changes in the HRQL dimensions in the course of child development [14,15,17]. The KINDL^R^ questionnaire consists of 24 items that assess six subscales: physical well-being, emotional well-being, self-esteem, family, friends, and everyday functioning (school) [17]. A total score of these six subscales is calculated after transforming the raw data on a scale ranging from 0 to 100. The Turkish version of the KINDL^R^ questionnaire was modified by Eser et al. [18] and the results of their study indicated that the Turkish Kid-KINDL is a reliable and factorially valid assessment of children’s HRQL.

### Statistical Analysis

The data were evaluated using SPSS 20.0 for Windows. Comparing the categorical variables of groups, nonparametric Kruskal-Wallis analysis was used. The Student t-test and Mann-Whitney U test were used when appropriate. Correlation and regression were used for determining the relationship between quality of life scores. Correlation was calculated with the Pearson correlation as a parametric method, while Spearman rank correlations (categorical variables) were calculated to evaluate relationships between changes in HRQL scores.

This study was approved by the Ethics Committee of the Dokuz Eylül University Faculty of Medicine (approval number: 2015/10-11).

## Results

The mean age of patients at the time of the study was 12.7±2.5 years and the mean age at diagnosis was 4.8±2.4 years. Forty-two patients (60%) were in the age group of 7-13 years and 28 patients (40%) were in the age group of 14-17 years. The study group represented 65% of the survivors treated at two centers with similar characteristics. The sociodemographic properties of the study group are given in Table 1. Median follow-up duration was 8.2 years after completion of chemotherapy (Table 2). The mean age of the siblings was 14.1±2.9 years. There was statistically no difference between the two groups in age or sex. Information about the treatment center, therapy type, risk group of patients, relapse history, and time after therapy is summarized in Table 2.

Twelve patients (17.1%) had chronic disease. Seven of them had endocrinological disease (obesity, insulin resistance, hypothyroidism), 4 had cardiac disease (2 hypertension, 2 minimal diastolic dysfunction), and 1 had epilepsy.

Statistically, no differences could be found among HRQL scores of survivors with respect to variables including sex, therapy type, risk group, time after completion of therapy, income status, having chronic illness, relapse history, and therapy center.

The mean HRQL scores of patients, siblings, and healthy and chronically ill children (children with chronic diseases such as asthma, obesity, and neurodermatitis) from self-reports and parent proxy reports are given in Table 3 [14,15,17]. There were statistically no differences between the scores of patient self-reports, sibling self-reports, and parent proxy reports. Total HRQL scores and the HRQL scores for physical well-being, emotional well-being, family, and social functioning of the patients and sibling self-reports and parent proxy reports were lower than the expected values for healthy and chronically ill children. The HRQL scores for everyday functioning (school) of the patients appeared to be lower than the expected values for healthy children.

When we investigated the HRQL scores of patient self-reports and sibling self-reports, we found no significant correlations between the two groups (physical well-being: r=0.266, p=0.245; emotional well-being: r=0.264, p=0.275; self-esteem: r=-0.159, p=0.503; friends: r=0.122, p=0.609; school: r=-0.016, p=0.948; family: r=0.354, p=0.125; and total score: r=0.312, p=0.136). When we investigated the HRQL scores of patient self-reports and parent proxy reports, we found moderate but significant positive correlations for all subscales (physical well-being: r=0.444, p=0.000; emotional well-being: r=0.331, p=0.005; self-esteem: r=0.244, p=0.042; friends: r=0.266, p=0.026; school: r=0.344, p=0.004; family: r=0.269, p=0.024; and total score: r=0.258, p=0.032).

## Discussion

Quality of life is negatively affected by the adverse effects of treatment in survivors of childhood leukemia [19,20,21]. In this study, we found no differences among HRQL subscale scores of ALL survivors with respect to variables including sex, therapy type, risk group, time after therapy, income status, having chronic illness, relapse history, and therapy center. Most of our patients were followed for more than 6 years after completion of therapy. A recent review of 22 studies representing 2073 children found that overall HRQL is reduced among children with ALL receiving treatment compared to healthy children, and HRQL was reported to improve over time after completion of therapy [20]. In the same review, inconsistent associations between clinical/demographic factors and HRQL outcomes were found; poor HRQL during ALL treatment appeared to be associated with intensive phases of chemotherapy, experiencing greater toxicity, corticosteroid therapy, older age, and female sex. High-risk patients who have undergone hematopoietic stem cell therapy may have poorer HRQL scores. In a recent study, it was demonstrated that HRQL and depression scores were significantly lower among survivors 2-5 years after treatment when compared to 6-10 years and 10 years or more [21]. Similar to our findings, Kanellopoulos et al. [19] and Sitaresmi et al. [22] reported that demographic characteristics, cancer, and treatment-related variables were not associated with poor HRQL in survivors. Identifying children at higher risk of these side effects and determining effective supportive care can improve HRQL outcomes [19,20,23,24].

Evaluation of HRQL in children is not easy [12,25,26]. Patients who were 7-17 years old were included in this study to obtain reliable results and appropriate KINDL^R^ questionnaires were used for child self-reports and parent proxy reports [14,15,17]. We found that total HRQL scores of the ALL survivors appeared to be lower than the expected values for healthy and chronically ill children in the literature [14,15,17]. A strong association between poor HRQL in survivors of pediatric ALL and depression, anxiety, insomnia, pain, and obesity was found in several studies [19,20,21,27,28,29,30]. As a remarkable finding of our study, the scores for physical well-being, emotional well-being, family, and social functioning of the patient and sibling self-reports and parent proxy reports also appeared to be lower than the expected values for healthy and chronically ill child self-reports and their parent proxy reports. Including siblings and healthy children for two different control groups would make our study results stronger. As a control group, we could have chosen healthy children, but their family functioning and sociocultural situations could show differences. The reduced HRQL scores for these subscales may be due to problematic family functioning, household size, or social and cultural impairment [19,30]. The diagnosis, treatment, and follow-up periods for ALL cause significant disruption to normal family life and this negative effect may continue for many years [19,20,21].

In our study the HRQL scores of patient self-reports and parent proxy reports showed moderate but significant positive correlations in all subscales. It has been established that parent perception is important and ideally should reflect the child self-report [31,32,33]. The parent’s perception may not always be consistent with the child’s self-report, with parents overestimating their children’s impairment [20,34,35,36]. Mothers were more likely to report better HRQL in their children than fathers during induction therapy for ALL [37]. Children who self-reported poorer quality of life had mothers who were more depressed; parents who reported poorer quality of life for their children reported more illness stressors and perceived their children as being more vulnerable [36]. Exploration of the reasons for differences may improve the parent-child relationship.

## Conclusion

The low HRQL scores of the patient and sibling self-reports and parent proxy reports showed that both ALL survivors and their families need help via psychological counseling programs. Identifying patients and families at risk and providing them psychological support may improve the HRQL for ALL survivors and their families.

## Figures and Tables

**Table 1:**
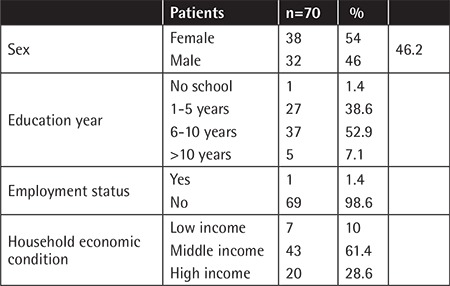
Sociodemographic data of the patients.

**Table 2:**
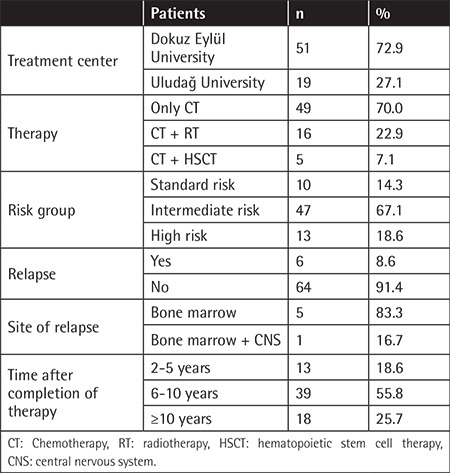
Information about patients’ treatment center, therapy type, risk group, relapse history, and time after completion of therapy.

**Table 3:**
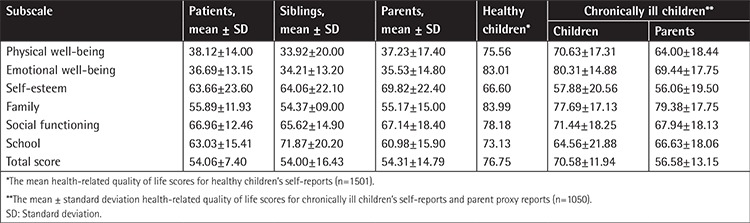
Health-related quality of life scores by patient self-reports, sibling self-reports, and parent proxy reports.
